# Medical Conditions Predictive of Self-Reported Poor Health: Retrospective Cohort Study

**DOI:** 10.2196/13018

**Published:** 2020-01-08

**Authors:** M Soledad Cepeda, Jenna Reps, David M Kern, Paul Stang

**Affiliations:** 1 Janssen Research & Development Titusville, NJ United States

**Keywords:** burden of illness, claims database studies, poor health

## Abstract

**Background:**

Identifying the medical conditions that are associated with poor health is crucial to prioritize decisions for future research and organizing care. However, assessing the burden of disease in the general population is complex, lengthy, and expensive. Claims databases that include self-reported health status can be used to assess the impact of medical conditions on the health in a population.

**Objective:**

This study aimed to identify medical conditions that are highly predictive of poor health status using claims databases.

**Methods:**

To determine the medical conditions most highly predictive of poor health status, we used a retrospective cohort study using 2 US claims databases. Subjects were commercially insured patients. Health status was measured using a self-report health status response. All medical conditions were included in a least absolute shrinkage and selection operator regression model to assess which conditions were associated with poor versus excellent health.

**Results:**

A total of 1,186,871 subjects were included; 61.64% (731,587/1,186,871) reported having excellent or very good health. The leading medical conditions associated with poor health were cancer-related conditions, demyelinating disorders, diabetes, diabetic complications, psychiatric illnesses (mood disorders and schizophrenia), sleep disorders, seizures, male reproductive tract infections, chronic obstructive pulmonary disease, cardiomyopathy, dementia, and headaches.

**Conclusions:**

Understanding the impact of disease in a commercially insured population is critical to identify subjects who may be at risk for reduced productivity and job loss. Claims database studies can measure the impact of medical conditions on the health status in a population and to assess changes overtime and could limit the need to collect prospective collection of information, which is slow and expensive, to assess disease burden. Leading medical conditions associated with poor health in a commercially insured population were the ones associated with high burden of disease such as cancer-related conditions, demyelinating disorders, diabetes, diabetic complications, psychiatric illnesses (mood disorders and schizophrenia), infections, chronic obstructive pulmonary disease, cardiomyopathy, and dementia. However, sleep disorders, seizures, male reproductive tract infections, and headaches were also part of the leading medical conditions associated with poor health that had not been identified before as being associated with poor health and deserve more attention.

## Introduction

Knowing which medical conditions are associated with perceived poor health is crucial to identify unmet needs and prioritize decisions for future research and interventions. However, assessing burden of disease in the general population is complex, lengthy, and expensive [1,2]. The Global Burden of Disease Study (GBD) created a framework for integrating and analyzing information on mortality and population health to compare the importance of diseases as measured by their impact on premature death and disability in different populations [3]. It requires assessing both the prevalence of each condition of interest and the impact of such conditions on a person’s overall health status, which often depends on collection of information that is not otherwise systematically collected in the larger population databases.

Claims databases contain data on millions of subjects that allow researchers to estimate the prevalence of a large number of medical conditions, including rare conditions that come to medical attention. Claims databases, however, usually lack information on self-reported outcomes needed to understand the impact of the medical conditions on overall health. This limitation can be overcome by linking a claims database with surveys that have information on health status and, unlike many electronic health record sources, are systematically collected in a defined population. The IBM MarketScan Health Risk Assessment (HRA) Database has self-reported health status information and can be linked to another IBM database—MarketScan Commercial Claims and Encounters (CCAE)—which contains data on health insurance claims of commercially insured individuals. This linkage allows researchers to efficiently study the burden of disease in a real-world setting in the employed population. Understanding the impact of disease in this population is critical to identify subjects who may be at risk of reduced productivity and job loss, a phenomenon that has been described extensively in the literature [4].

The impact of disease can be measured by self-reported health status, which in the HRA is captured in a single question: “How would you describe your overall health?” This single question has long been used to measure health status and health-related quality of life in national surveys or as part of multidimensional health status measures as it has been shown to be strongly associated with productivity [5], health care utilization, and mortality [6-10].

We sought to determine, in a commercially insured population, the medical conditions most highly predictive of poor health status.

## Methods

### Data Sources

To determine the medical conditions that are associated with self-rated poor health in a commercially insured population, we conducted a retrospective cohort study using 2 linked databases: CCAE and HRA.

The CCAE database represents data from individuals enrolled in US employer-sponsored insurance health plans. The data include adjudicated health insurance claims (ie, inpatient, outpatient, and outpatient pharmacy) as well as enrollment data from large employers and health plans who provide private health care coverage to employees, their spouses, and dependents. The database has inpatient and outpatient medical claims and medical diagnoses that are coded using the International Classification of Diseases (ICD) system ICD-9 or ICD-10.

The HRA database contains self-reported health-related behavioral data from surveys of employees of large US corporations and health plans. These questionnaires are administered as part of corporate health and wellness programs and are designed to help employees understand their own health risks and how they may be able to mitigate the risks. Participation is voluntary, although employers often provide incentives such as a credit toward the employee’s share of medical premiums for completion of the survey.

### Health Status

To determine the health status of the responder, we used the answer to the single question: “Over the past 6 months, how would you describe your overall health?” The 5 potential responses were excellent, very good, good, fair, and poor.

This single question is simple, easy to understand, [11] reliable [12], and, as mentioned above, has been shown to be strongly associated with productivity [5], health care utilization, and mortality [6-9].

We included survey responses from 2008 to 2016. When subjects responded to the survey in more than 1 year, we selected the most recent response. The date of the survey was considered the index date.

### Medical Conditions

Diagnosis codes from medical claims occurring within the 6 months preceding the patients’ survey date were included as candidate predictors of self-reported health. To group medical conditions, we used the Medical Dictionary for Regulatory Activities vocabulary (MedDRA). MedDRA is a rich and highly specific standardized medical terminology created to facilitate sharing of regulatory information internationally for medical products. It was developed in the late 1990s by the International Council for Harmonization of Technical Requirements for Pharmaceuticals for Human Use. The advantage of this vocabulary is that the terminology is hierarchically arranged from very specific to very general. We used the High-Level Group level to group the conditions. We used existing mappings of ICD-9 or ICD-10 codes to obtain MedDRA groups [13]. For example, the atrial fibrillation ICD-10 code (I48) is mapped to atrial fibrillation, which then rolls up to the High-Level Group cardiac arrhythmias.

### Analysis

We built a least absolute shrinkage and selection operator (LASSO) logistic regression model [14] to assess which conditions were associated with poor versus excellent health at the time the subject responded to the survey. LASSO regression is similar to standard logistic regression except it adds a model complexity penalty to “shrink” the coefficients toward 0. Some of the coefficients are completely shrunk to 0, and therefore, LASSO reduces the number of variables used in the final model. The advantages are that it effectively does variable selection during model training, which reduces that occurrence of model overfitting and often results in a more parsimonious model. It is able to find the strongest predictors of having poor versus excellent health. We used the LASSO results to rank the medical conditions associated with poor outcomes.

We also performed a traditional logistic regression to include only MedDRA groups that were not highly correlated with one another (r<0.70), and the results were consistent with the LASSO regression and thus, are not reported.

The regression model included medical conditions recorded in the claims data during the 6 months preceding the index date to reflect the same 6-month timeframe that is incorporated into the health status question. We included 260 medical conditions (MedDRA High Level Groups; Multimedia Appendix 1), and the outcome of interest was self-reported poor health status. The reference group included individuals self-reporting excellent health.

Odds ratios and 95% CIs were calculated using the beta coefficients and SEs of the logistic regression model and represent the independent association of each condition adjusted for the presence of all other conditions included in the model. We report the odds ratios from the logistic regression because the coefficients from the LASSO regression are shrunk and should not be interpreted as odd ratios. In addition, we present the prevalence of the conditions in subjects with and without the outcome of interest.

### Validation

To validate the study findings, the model was trained using 3-fold cross validation on 75% of the data (training sample), and the study findings were validated on the remaining 25% of the data (test sample).

To assess the performance of the LASSO regression model, we calculated area under the curve (AUC) using the test sample. The AUC is a measure that quantifies the ability of the model to discriminate between subjects with and without the outcome [15]. The higher the AUC, the better the model discriminates between the subject with and without poor health.

### Generalizability

To assess whether the results of the study generalize to a broader population, we compared the survey responders with the general commercially insured population.

We took a random sample of primary beneficiaries in the CCAE database of the same size as the survey responders stratified by year, and we required that the subjects be in the CCAE database at least 6 months before the index date. The index date for subjects who did not respond to the survey was a randomly selected date within the same calendar year.

We calculated age, number of distinct medical conditions, and number of visits to the health care system 6 months before the index date and the Charlson comorbidity index score [16] to further characterize the population for comparison. As comorbidities are major determinants of patient health status, we included the Charlson Index, which is a weighted sum of the presence of 19 medical conditions; each condition is assigned a weight from 1 to 6, with higher weights indicating greater severity and higher risk of mortality.

## Results

### Study Population

A total of 1,415,789 subjects answered the health status question, of whom 1,186,871 met the requirements of being in the CCAE database for at least 6 months before the day they responded to the survey. A total of 61.64% (731,587/1,186,871) of the responders reported having excellent or very good health; see Table 1.

The survey responders did not differ substantially from the subjects in the CCAE database with regard to age and gender. However, survey responders had more visits to the health care system (5.0 vs 3.3) and more medical conditions (3.8 vs 3.1) than the remaining subjects in the CCAE database; see Table 2.

**Table 1:** Health status of survey responders (N=1,186,871).

Self-reported health status	Survey responders, n (%)
Excellent health	239,734 (20.20)
Very good	491,845 (41.44)
Good health	365,083 (30.76)
Fair health	77,997 (6.57)
Poor health	12,212 (1.03)

**Table 2:** Characteristic of the survey responders and the source population.

Characteristic		Random sample of employees in CCAE^a^ (N=1,186,871)	All survey responders (N=1,186,871)	Subjects reporting excellent health (N=239,734)	Subjects reporting poor health (N=12,212)
**Sex, n (%)**					
	Male	616,901 (51.97)	623,668 (52.54)	128,748 (53.70)	5758 (47.15)
	Female	569,970 (48.02)	563,203 (47.45)	110,986 (46.29)	6454 (52.84)
Age (years), mean (SD)		42.5 (12.35)	44.3 (11.43)	44.4 (11.64)	43.6 (11.56)
Charlson Index, mean (SD)		0.39 (1.63)	0.69 (1.1)	0.49 (1.32)	1.3 (2.51)
Distinct number of conditions 6 months preindex, mean (SD)		3.1 (5.72)	3.8 (5.28)	2.9 (4.12)	7.1 (9.41)
Number of visits 6 months preindex, mean (SD)		3.3 (7.23)	5.0 (8.02)	4.0 (6.34)	9.5 (15.12)

^a^CCAE: Commercial Claims and Encounters.

The outcome was initially defined as having a self-reported fair or poor health status, and these subjects were compared with subjects who reported having good, very good, or excellent health. The AUC model that used this delineation was 0.66. To improve the discrimination of the model, we implemented a different threshold where subjects who reported poor health were compared with subjects who reported excellent health. The performance of model improved with an AUC of 0.73.

A total of 251,892 subjects were included in the regression model that compared subjects who reported poor health (n=12,212) with subjects who reported excellent health (n=239,734). Subjects with poor health had more diagnosed conditions, more prior visits, and a higher Charlson index score than subjects with excellent health; see Table 2.

### Leading Medical Conditions

The leading medical conditions that were associated with poor health were cancer-related conditions, demyelinating disorders, diabetes/diabetic complications, psychiatric illnesses (mood disorders and schizophrenia), sleep disorders, seizures, male reproductive tract infections, chronic obstructive pulmonary disease, cardiomyopathy, dementia, and headaches (Table 3). Substance use disorders, diabetes, mood disorders, sleep disorders, and obstructive pulmonary disease were the most prevalent among subjects with poor health. The association of all medical conditions assessed and their prevalence in subjects with poor and excellent health are listed in Multimedia Appendix 1.

**Table 3:** Leading medical conditions associated with poor health and their prevalence in subjects with poor or excellent health.

Medical condition	Prevalence in subjects with poor health, %	Prevalence in subjects with excellent health, %	Adjusted odds ratio (OR) from logistic regression model^a^ (95% CI)
Metastases	1.56	0.05	7.15 (4.92-10.39)
Demyelinating disorders	0.66	0.08	3.16 (2.32-4.29)
Skeletal neoplasms malignant and unspecified	0.74	0.06	2.24 (1.37-3.68)
Glucose metabolism disorders	15.03	2.82	2.55 (1.98-3.29)
Diabetic complications	4.26	0.33	2.11 (1.79-2.48)
Manic and bipolar mood disorders and disturbances	1.55	0.24	1.98 (1.62-2.43)
Neoplasm-related morbidities	0.40	0.05	2.03 (1.28-3.22)
Sleep disturbances	10.79	2.53	1.93 (1.79-2.09)
Hepatobiliary neoplasms	0.55	0.02	1.99 (1.14-3.46)
Male reproductive tract infections and inflammations	0.49	0.36	1.73 (1.27-2.38)
Seizures	1.03	0.21	1.81 (1.41-2.32)
Increased intracranial pressure and hydrocephalus	0.17	0.02	2.09 (1.14-3.84)
Heart failures	3.49	0.51	1.69 (1.43-2.00)
Hematopoietic neoplasms (excluding leukemias and lymphomas)	3.77	0.93	1.65 (1.33-2.05)
Lymphomas non-Hodgkin T-cell	0.18	0.03	2.43 (1.17-5.04)
Gastrointestinal hemorrhages	1.62	0.63	1.47 (1.19-1.81)
Depressed mood disorders and disturbances	10.97	2.38	1.71 (1.45-2.01)
Bronchial disorders (excluding neoplasms)	10.63	3.58	1.60 (1.47-1.74)
Dementia and amnestic conditions	0.74	0.11	1.61 (1.17-2.21)
Lymphatic vessel disorders	0.45	0.05	2.20 (1.37-3.54)
Plasma cell neoplasms	0.29	0.04	1.93 (1.16-3.20)
Schizophrenia and other psychotic disorders	0.87	0.17	1.43 (1.10-1.85)
Substance-related disorders	22.58	8.24	1.52 (1.38-1.66)
Myocardial disorders	2.00	0.31	1.62 (1.31-1.98)
Headaches	7.75	2.73	1.26 (1.15-1.38)

^a^The odds ratios come from the logistic regression model that had all medical conditions with correlations <0.7.

## Discussion

### Principal Findings

Cancer-related conditions, demyelinating disorders, diabetes/diabetic complications, psychiatric illnesses (mood disorders and schizophrenia), sleep disorders, seizures, male reproductive tract infections, chronic obstructive pulmonary disease, cardiomyopathy, dementia, and headaches were the leading medical conditions associated with poor health.

Many of the medical conditions that had a strong association with poor health in our commercially insured population are similar to the conditions identified as the ones that affect the health of the general population using the GBD framework [1,2]. For example, cancer, diabetes, and mood disorders are the leading medical conditions associated with disability and mortality in the GBD study, and in our study, they were also some among the most predictive of having self-reported poor health status. This was of particular interest as the GBD made extensive use of studies using screening questionnaires (eg, for mood, which would identify sufferers regardless of whether they sought medical attention), whereas our analysis was based on interactions with the health care system. Using claims data for these analyses comes with the conceptual acceptance that for many conditions such as diabetes and cancer, it is unlikely that there are undetected “cases” in the population, whereas for disorders such as mood or anxiety, only a portion of those affected seek care and are adequately identified. Nesting our analysis in an employed population with access to insurance also tempers the potential impact of access to care that is associated with health care–seeking behavior differences by reimbursement coverage.

Of interest, there are some notable differences between our findings and the GBD rankings. For example, stroke was not one of our top 25 conditions associated with poor health, but stroke has been identified as one the top 10 conditions with substantial impact on health measured by mortality or disability-adjusted life-years [1,2]. One reason for these differences may be because of the populations being studied. Our study included employed individuals with commercial insurance who completed a survey, and thus, conditions that are acute and highly fatal or debilitating—such as stroke—or those that are more likely in an older population may not be well represented in a comparatively healthy workforce population (often referred to as the Health Worker effect). This is further reflected when comparing results with those from the general US population, as approximately 10% of the population self-report poor health status [17], but in our population, only 1% did, which may also reflect a relatively younger population. A second reason may be differences in how burden of disease was measured. For example, stroke drops from the 2nd position in the ranking for mortality to the 17th position when years lived with disability is used to assess the burden of disease. In this study, we used the magnitude of the association of the condition with poor health.

We also found some conditions at the top of our list for their association with poor health that are not in the top 25 conditions when the GBD framework is used. Focusing on a commercially insured population allowed us to identify conditions that are specifically relevant for that population and may otherwise be overlooked. This is important given a major health policy objective is to maintain a healthy workforce by reducing the impact of disease on disablement and productivity. One of the important predictors of poor health that have not been previously identified is sleep disorders. Sleep disorders are not among the 25 leading diseases that affect life expectancy or disability in the United States or globally [1,2]. Our finding adds to the body of evidence on the negative impact of sleep loss on health outcomes. Subjects who sleep less than or equal to 6 hours and subjects with insomnia not only have higher BMI but also have more cardiovascular problems [18] and increased rates of death [19]. Another condition predictive of poor health was reproductive tract infections, which includes chronic prostatitis. Chronic prostatitis affects men of all ages and demographics, and this study also confirms the substantial impact it has on quality of life [20].

This study also confirms the disease burden of infrequent conditions such as multiple sclerosis, which too was not on the top 25 conditions in the GBD study. Multiple sclerosis is a rare progressive chronic progressive autoimmune neurological disease [21]. Despite the availability of treatments, it is a leading predictor of poor health.

In this study, we are reporting the results of a comparison between subjects who reported poor health with subjects who reported excellent health because this model performed better than the model in which we grouped subjects who had poor and fair health and compared them with subjects who reported having good, very good, or excellent health. Studies that have assessed the reliability of the single self-reported health status have found that a large number of subjects inconsistently report their ratings when self-assessing health [22]. Most subjects who change ratings do it by only 1 category. So, the comparison between subjects who report poor health versus subjects who report excellent status, a comparison of the extreme responses, is likely to have less misclassification, and therefore, the model can better discriminate between the 2 groups.

### Study Limitations

As mentioned above, this study used administrative medical claims to find the leading medical conditions associated with self-report of poor health. These medical conditions were identified through medical claims data, which are generated for administrative and reimbursement, not for research purposes, so the presence of a claim with a specific diagnosis does not necessarily indicate the presence of that condition. This misclassification, although it will not affect the ranking, would lead to underestimation of the association with poor health. In addition, the population studied is a commercially insured population that is healthy enough to work, so the prevalence of conditions that occur mainly in a nonworking or elderly population are likely to be underestimated.

### Conclusions

Understanding the impact of disease in commercially insured subjects is critical to identify subjects who may be at risk of reduced productivity and job loss. Claims databases that have self-reported health status provide a very efficient and valid way to provide an overview of the impact of medical conditions on the health in a population and to assess changes overtime. Prospective collection of information is slow and expensive; however, this expensive approach could be tailored and focused to supplement the information that can be obtained from claims or similar databases. We found that leading medical conditions associated with poor health in a commercially insured population were the ones associated with high burden of disease in the World Health Organization GBD study such as cancer-related conditions, demyelinating disorders, diabetes/diabetic complications, psychiatric illnesses (mood disorders and schizophrenia), infections, chronic obstructive pulmonary disease, cardiomyopathy, and dementia. However, sleep disorders, seizures, male reproductive tract infections, and headaches were also part of the leading medical conditions associated with poor health that had not been identified before as being associated with poor health and deserve more attention.

## Appendix

Multimedia Appendix 1 Prevalence of each of the 260 medical conditions considered in the logistic regression model and their association with poor versus excellent health.

## Abbreviations

AUC: area under the curve
CCAE: Commercial Claims and Encounters
GBD: Global Burden of Disease Study
HRA: Health Risk Assessment
ICD: International Classification of Diseases
LASSO: least absolute shrinkage and selection operator
MedDRA: Medical Dictionary for Regulatory Activities
