# Supplementary material for: Medical Conditions Predictive of Self-Reported Poor Health: Retrospective Cohort Study
Source: JMIR Public Health Surveill. 2020 Jan 8;6(1):e13018. doi: 10.2196/13018 (PMC6996740; doi:10.2196/13018)
Supplement: Multimedia Appendix 1 [file publichealth_v6i1e13018_app1.docx]

**Additional file 1**

**Prevalence of each of the 260 medical conditions considered in the logistic regression model and their association with poor versus excellent health**

| **Condition** | **Prevalence in subjects with poor health** | **Prevalence in subjects with excellent health** | **OR from fully adjusted logistic regression model** | **Lower 95% CI** | **Upper 95% CI** |
| --- | --- | --- | --- | --- | --- |
| Musculoskeletal and connective tissue disorders NEC | 51.4% | 37.8% | 0.95 | 0.90 | 1.01 |
| Respiratory disorders NEC | 37.5% | 18.4% | 1.35 | 1.26 | 1.44 |
| Infections - pathogen unspecified | 36.0% | 22.8% | 1.13 | 1.05 | 1.21 |
| Metabolism disorders NEC | 35.0% | 17.4% | 0.96 | 0.90 | 1.02 |
| Vascular disorders NEC | 32.0% | 13.2% | 1.65 | 1.56 | 1.75 |
| Arteriosclerosis, stenosis, vascular insufficiency and necrosis | 31.3% | 12.4% | NA |  |  |
| Protein and amino acid metabolism disorders NEC | 28.0% | 12.4% | NA |  |  |
| Joint disorders | 27.2% | 15.4% | 1.14 | 1.06 | 1.23 |
| Gastrointestinal conditions NEC | 26.1% | 13.7% | 1.11 | 1.02 | 1.21 |
| Neurological disorders NEC | 23.6% | 8.8% | 1.14 | 1.03 | 1.26 |
| Skin and subcutaneous tissue disorders NEC | 22.8% | 16.7% | 1.16 | 1.05 | 1.27 |
| Substance use disorders | 22.6% | 8.2% | 1.52 | 1.38 | 1.66 |
| Vascular hypertensive disorders | 22.5% | 7.6% | NA |  |  |
| Viral infectious disorders | 22.0% | 12.7% | 1.02 | 0.95 | 1.10 |
| Respiratory tract infections | 21.7% | 12.8% | NA |  |  |
| Reproductive tract disorders NEC | 21.5% | 14.2% | 1.03 | 0.95 | 1.12 |
| Endocrine and glandular disorders NEC | 21.5% | 8.0% | 1.01 | 0.92 | 1.11 |
| Allergic conditions | 21.4% | 11.5% | 0.96 | 0.88 | 1.04 |
| Epidermal and dermal conditions | 19.9% | 18.2% | 0.97 | 0.90 | 1.05 |
| Connective tissue disorders (excl congenital) | 19.5% | 10.4% | 1.10 | 1.01 | 1.20 |
| Genitourinary tract disorders NEC | 18.0% | 11.0% | NA |  |  |
| Spinal cord and nerve root disorders | 17.4% | 5.7% | 1.38 | 1.24 | 1.55 |
| Tissue disorders NEC | 17.4% | 14.8% | 0.97 | 0.87 | 1.08 |
| Lipid metabolism disorders | 17.3% | 9.9% | NA |  |  |
| Mood disorders and disturbances NEC | 16.8% | 5.0% | 1.24 | 1.06 | 1.45 |
| Injuries NEC | 16.5% | 10.4% | 0.97 | 0.90 | 1.05 |
| Metabolic and nutritional disorders congenital | 16.5% | 4.3% | NA |  |  |
| Tendon, ligament and cartilage disorders | 15.1% | 8.4% | NA |  |  |
| Glucose metabolism disorders (incl diabetes mellitus) | 15.0% | 2.8% | 2.55 | 1.98 | 3.29 |
| Inborn errors of metabolism | 14.7% | 2.9% | 1.18 | 0.92 | 1.52 |
| Cornification and dystrophic skin disorders | 14.1% | 14.3% | NA |  |  |
| Bone disorders (excl congenital and fractures) | 12.6% | 6.5% | 0.99 | 0.91 | 1.08 |
| Appetite and general nutritional disorders | 12.6% | 3.8% | 1.64 | 1.53 | 1.76 |
| Upper respiratory tract disorders (excl infections) | 12.3% | 8.6% | 0.66 | 0.59 | 0.74 |
| Miscellaneous and site unspecified neoplasms malignant and unspecified | 12.3% | 11.3% | NA |  |  |
| Immune disorders NEC | 11.8% | 5.7% | 1.12 | 1.01 | 1.25 |
| Musculoskeletal and connective tissue neoplasms | 11.2% | 10.6% | 0.94 | 0.83 | 1.06 |
| Sleep disorders and disturbances | 11.1% | 2.7% | NA |  |  |
| Miscellaneous and site unspecified neoplasms benign | 11.1% | 11.4% | NA |  |  |
| Depressed mood disorders and disturbances | 11.0% | 2.4% | 1.71 | 1.45 | 2.01 |
| Sleep disturbances (incl subtypes) | 10.8% | 2.5% | 1.93 | 1.79 | 2.09 |
| Peripheral neuropathies | 10.7% | 3.1% | NA |  |  |
| Musculoskeletal and connective tissue deformities (incl intervertebral disc disorders) | 10.6% | 4.4% | 1.17 | 1.06 | 1.30 |
| Bronchial disorders (excl neoplasms) | 10.6% | 3.6% | 1.60 | 1.47 | 1.74 |
| Thoracic disorders (excl lung and pleura) | 10.5% | 5.7% | 0.89 | 0.79 | 0.99 |
| Coronary artery disorders | 9.9% | 3.0% | 1.31 | 1.20 | 1.43 |
| Gastrointestinal motility and defaecation conditions | 9.3% | 3.6% | 1.12 | 1.02 | 1.24 |
| Bone and joint injuries | 9.2% | 5.7% | 0.86 | 0.76 | 0.98 |
| Anxiety disorders and symptoms | 9.1% | 3.2% | 0.98 | 0.88 | 1.08 |
| Muscle disorders | 8.9% | 3.4% | 1.21 | 1.11 | 1.32 |
| Soft tissue neoplasms benign | 8.6% | 9.4% | NA |  |  |
| Vascular haemorrhagic disorders | 7.9% | 4.0% | 0.90 | 0.81 | 1.01 |
| Headaches | 7.8% | 2.7% | 1.26 | 1.15 | 1.38 |
| Skin and subcutaneous tissue infections and infestations | 7.7% | 4.0% | 1.13 | 1.00 | 1.26 |
| Eye disorders NEC | 7.7% | 6.5% | 0.96 | 0.83 | 1.10 |
| Autoimmune disorders | 7.5% | 2.4% | 1.31 | 1.19 | 1.44 |
| Thyroid gland disorders | 7.3% | 4.4% | NA |  |  |
| Electrolyte and fluid balance conditions | 7.0% | 1.7% | 1.27 | 1.12 | 1.43 |
| Central nervous system infections and inflammations | 6.8% | 2.8% | 0.90 | 0.77 | 1.06 |
| Vulvovaginal disorders (excl infections and inflammations) | 6.0% | 4.4% | 0.83 | 0.69 | 1.01 |
| Renal disorders (excl nephropathies) | 5.9% | 1.6% | 1.51 | 1.31 | 1.74 |
| Oral soft tissue conditions | 5.8% | 3.5% | 1.22 | 1.06 | 1.40 |
| Leukaemias | 5.8% | 2.1% | 1.18 | 1.05 | 1.33 |
| Bacterial infectious disorders | 5.6% | 2.2% | 1.21 | 1.07 | 1.37 |
| Anaemias nonhaemolytic and marrow depression | 5.4% | 1.7% | 1.07 | 0.94 | 1.21 |
| Uterine, pelvic and broad ligament disorders | 5.3% | 3.3% | 1.22 | 1.01 | 1.46 |
| Vitamin related disorders | 5.3% | 2.7% | NA |  |  |
| Encephalopathies | 5.2% | 1.4% | 0.76 | 0.66 | 0.88 |
| Malignant and unspecified neoplasms gastrointestinal NEC | 5.2% | 3.0% | 1.22 | 0.97 | 1.53 |
| Haematological disorders NEC | 5.0% | 1.4% | NA |  |  |
| Nephropathies | 4.9% | 1.1% | NA |  |  |
| Aural disorders NEC | 4.8% | 2.9% | 1.15 | 0.95 | 1.39 |
| Decreased and nonspecific blood pressure disorders and shock | 4.7% | 1.4% | 1.12 | 0.97 | 1.28 |
| Gastrointestinal inflammatory conditions | 4.7% | 1.6% | 1.29 | 1.14 | 1.47 |
| Cutaneous neoplasms benign | 4.6% | 7.6% | 0.58 | 0.50 | 0.67 |
| Skin neoplasms malignant and unspecified | 4.6% | 7.3% | NA |  |  |
| Skin appendage conditions | 4.5% | 3.9% | 0.82 | 0.73 | 0.92 |
| Spleen, lymphatic and reticuloendothelial system disorders | 4.4% | 3.7% | 0.81 | 0.67 | 0.98 |
| Ocular infections, irritations and inflammations | 4.4% | 3.7% | 0.81 | 0.70 | 0.93 |
| Diabetic complications | 4.3% | 0.3% | 2.11 | 1.79 | 2.48 |
| Anterior eye structural change, deposit and degeneration | 4.2% | 3.6% | NA |  |  |
| Soft tissue neoplasms malignant and unspecified | 4.1% | 3.3% | 0.88 | 0.75 | 1.03 |
| Cardiac arrhythmias | 4.0% | 1.3% | 1.01 | 0.88 | 1.16 |
| Ocular structural change, deposit and degeneration NEC | 3.8% | 2.9% | 0.74 | 0.63 | 0.86 |
| Haematopoietic neoplasms (excl leukaemias and lymphomas) | 3.8% | 0.9% | 1.65 | 1.33 | 2.05 |
| Hepatic and hepatobiliary disorders | 3.8% | 0.9% | 1.35 | 1.16 | 1.57 |
| Sexual function and fertility disorders | 3.5% | 2.4% | 1.08 | 0.87 | 1.35 |
| Heart failures | 3.5% | 0.5% | 1.69 | 1.43 | 2.00 |
| Endocrine disorders of gonadal function | 3.5% | 2.3% | 1.07 | 0.88 | 1.30 |
| Gastrointestinal neoplasms malignant and unspecified | 3.3% | 1.6% | NA |  |  |
| Respiratory and mediastinal neoplasms malignant and unspecified | 3.3% | 3.5% | NA |  |  |
| Exposures, chemical injuries and poisoning | 3.2% | 2.4% | 1.02 | 0.89 | 1.16 |
| Menstrual cycle and uterine bleeding disorders | 3.2% | 2.3% | 1.16 | 0.99 | 1.36 |
| Fractures | 3.2% | 1.4% | 0.94 | 0.78 | 1.12 |
| Neuromuscular disorders | 3.2% | 0.9% | 1.16 | 1.01 | 1.34 |
| Central nervous system vascular disorders | 3.0% | 0.8% | 0.93 | 0.78 | 1.10 |
| Lower respiratory tract disorders (excl obstruction and infection) | 3.0% | 0.5% | 1.38 | 1.15 | 1.65 |
| Fungal infectious disorders | 2.9% | 1.6% | 1.01 | 0.86 | 1.18 |
| Breast disorders | 2.7% | 2.5% | 0.78 | 0.66 | 0.93 |
| Iron and trace metal metabolism disorders | 2.7% | 0.8% | 1.13 | 0.94 | 1.36 |
| Synovial and bursal disorders | 2.7% | 1.6% | 0.94 | 0.82 | 1.08 |
| Gastrointestinal infections | 2.6% | 1.0% | 0.92 | 0.78 | 1.08 |
| Urethral disorders (excl calculi) | 2.6% | 1.3% | 1.00 | 0.86 | 1.17 |
| Female reproductive tract infections and inflammations | 2.5% | 1.8% | 1.10 | 0.91 | 1.31 |
| Middle ear disorders (excl congenital) | 2.4% | 1.3% | 1.18 | 0.95 | 1.46 |
| Reproductive neoplasms female benign | 2.4% | 1.5% | 0.94 | 0.73 | 1.20 |
| Adjustment disorders (incl subtypes) | 2.4% | 1.5% | 0.77 | 0.67 | 0.89 |
| Venous varices | 2.3% | 1.7% | 0.87 | 0.73 | 1.05 |
| Cognitive and attention disorders and disturbances | 2.3% | 1.0% | 0.84 | 0.71 | 0.99 |
| Eye disorders congenital | 2.2% | 1.6% | NA |  |  |
| Gastrointestinal neoplasms benign | 2.2% | 1.6% | NA |  |  |
| Benign neoplasms gastrointestinal | 2.2% | 1.6% | 0.65 | 0.52 | 0.83 |
| Vision disorders | 2.1% | 1.2% | 1.19 | 1.00 | 1.41 |
| Myocardial disorders | 2.0% | 0.3% | 1.62 | 1.31 | 1.98 |
| Embolism and thrombosis | 2.0% | 0.4% | 1.30 | 1.05 | 1.60 |
| Respiratory tract neoplasms | 1.9% | 0.5% | NA |  |  |
| Congenital and hereditary disorders NEC | 1.8% | 0.9% | 1.36 | 1.08 | 1.72 |
| White blood cell disorders | 1.8% | 0.5% | 0.86 | 0.68 | 1.07 |
| Ovarian and fallopian tube disorders | 1.8% | 0.9% | 1.22 | 0.96 | 1.57 |
| Prostatic disorders (excl infections and inflammations) | 1.7% | 1.5% | 0.49 | 0.41 | 0.60 |
| Abdominal hernias and other abdominal wall conditions | 1.7% | 0.8% | 0.74 | 0.61 | 0.89 |
| Musculoskeletal and connective tissue disorders congenital | 1.7% | 0.8% | NA |  |  |
| Gastrointestinal vascular conditions | 1.7% | 1.3% | NA |  |  |
| Pleural disorders | 1.7% | 0.2% | 1.07 | 0.83 | 1.38 |
| Testicular and epididymal disorders | 1.7% | 1.1% | 0.68 | 0.52 | 0.88 |
| External ear disorders (excl congenital) | 1.6% | 1.3% | 0.76 | 0.62 | 0.94 |
| Reproductive neoplasms female malignant and unspecified | 1.6% | 1.2% | 0.79 | 0.58 | 1.06 |
| Cranial nerve disorders (excl neoplasms) | 1.6% | 0.9% | 0.94 | 0.75 | 1.18 |
| Gastrointestinal haemorrhages NEC | 1.6% | 0.6% | 1.47 | 1.19 | 1.81 |
| Cardiac valve disorders | 1.6% | 0.6% | 0.73 | 0.59 | 0.90 |
| Metastases | 1.6% | 0.0% | 7.15 | 4.92 | 10.39 |
| Bladder and bladder neck disorders (excl calculi) | 1.5% | 0.7% | NA |  |  |
| Manic and bipolar mood disorders and disturbances | 1.5% | 0.2% | 1.98 | 1.62 | 2.43 |
| Urolithiases | 1.5% | 0.6% | 0.96 | 0.77 | 1.19 |
| Anal and rectal conditions NEC | 1.4% | 0.8% | 1.12 | 0.91 | 1.38 |
| Bone, calcium, magnesium and phosphorus metabolism disorders | 1.4% | 0.7% | 0.61 | 0.49 | 0.76 |
| Diverticular disorders | 1.4% | 0.8% | 0.84 | 0.69 | 1.01 |
| Sexual dysfunctions, disturbances and gender identity disorders | 1.4% | 1.0% | 0.80 | 0.61 | 1.06 |
| Glaucoma and ocular hypertension | 1.4% | 1.4% | 0.71 | 0.58 | 0.86 |
| Movement disorders (incl parkinsonism) | 1.3% | 0.3% | 1.25 | 1.01 | 1.55 |
| Pregnancy, labour, delivery and postpartum conditions | 1.3% | 2.0% | 0.69 | 0.55 | 0.87 |
| Inner ear and VIIIth cranial nerve disorders | 1.3% | 0.6% | 1.06 | 0.84 | 1.34 |
| Reproductive neoplasms male malignant and unspecified | 1.3% | 1.0% | NA |  |  |
| Bile duct disorders | 1.2% | 0.3% | 1.13 | 0.90 | 1.41 |
| Coagulopathies and bleeding diatheses (excl thrombocytopenic) | 1.2% | 0.3% | 1.10 | 0.78 | 1.57 |
| Gastrointestinal ulceration and perforation | 1.2% | 0.3% | 1.41 | 1.10 | 1.81 |
| Menopause related conditions | 1.1% | 1.4% | 0.51 | 0.42 | 0.62 |
| Peritoneal and retroperitoneal conditions | 1.1% | 0.2% | 0.94 | 0.72 | 1.23 |
| Breast neoplasms malignant and unspecified (incl nipple) | 1.1% | 0.6% | 1.45 | 1.11 | 1.90 |
| Purine and pyrimidine metabolism disorders | 1.0% | 0.4% | 1.31 | 1.05 | 1.63 |
| Gallbladder disorders | 1.0% | 0.3% | NA |  |  |
| Seizures (incl subtypes) | 1.0% | 0.2% | 1.81 | 1.41 | 2.32 |
| Ancillary infectious topics | 1.0% | 0.4% | 0.83 | 0.64 | 1.07 |
| Mental impairment disorders | 0.9% | 0.2% | NA |  |  |
| Hearing disorders | 0.9% | 0.7% | 0.77 | 0.59 | 1.00 |
| Immunodeficiency syndromes | 0.9% | 0.3% | 1.01 | 0.73 | 1.41 |
| Retina, choroid and vitreous haemorrhages and vascular disorders | 0.9% | 0.2% | 1.13 | 0.84 | 1.52 |
| Structural brain disorders | 0.9% | 0.2% | 1.12 | 0.78 | 1.61 |
| Schizophrenia and other psychotic disorders | 0.9% | 0.2% | 1.43 | 1.10 | 1.85 |
| Neurological disorders of the eye | 0.8% | 0.5% | 0.76 | 0.54 | 1.06 |
| Acid-base disorders | 0.8% | 0.1% | 1.08 | 0.75 | 1.56 |
| Penile and scrotal disorders (excl infections and inflammations) | 0.8% | 0.6% | 0.84 | 0.64 | 1.11 |
| Platelet disorders | 0.8% | 0.2% | 0.89 | 0.58 | 1.37 |
| Vascular inflammations | 0.8% | 0.2% | 0.86 | 0.63 | 1.17 |
| Nervous system neoplasms malignant and unspecified NEC | 0.8% | 0.2% | 1.56 | 1.04 | 2.33 |
| Cervix disorders (excl infections and inflammations) | 0.8% | 0.7% | 0.72 | 0.56 | 0.94 |
| Pulmonary vascular disorders | 0.8% | 0.1% | 1.24 | 0.88 | 1.75 |
| Pigmentation disorders | 0.8% | 1.6% | 0.54 | 0.42 | 0.68 |
| Dementia and amnestic conditions | 0.7% | 0.1% | 1.61 | 1.17 | 2.21 |
| Skeletal neoplasms malignant and unspecified | 0.7% | 0.1% | 2.24 | 1.37 | 3.68 |
| Neoplastic and ectopic endocrinopathies | 0.7% | 0.2% | 1.19 | 0.83 | 1.72 |
| Endocrine neoplasms malignant and unspecified | 0.7% | 0.2% | NA |  |  |
| Lymphomas NEC | 0.7% | 0.2% | 1.19 | 0.76 | 1.86 |
| Gastrointestinal stenosis and obstruction | 0.7% | 0.2% | 0.98 | 0.72 | 1.34 |
| Parathyroid gland disorders | 0.7% | 0.2% | 1.48 | 1.06 | 2.07 |
| Demyelinating disorders | 0.7% | 0.1% | 3.16 | 2.32 | 4.29 |
| Malabsorption conditions | 0.6% | 0.2% | 0.82 | 0.62 | 1.09 |
| Cardiac and vascular disorders congenital | 0.6% | 0.3% | 0.52 | 0.36 | 0.75 |
| Exocrine pancreas conditions | 0.6% | 0.1% | 1.42 | 0.98 | 2.07 |
| Angioedema and urticaria | 0.6% | 0.4% | 0.85 | 0.63 | 1.15 |
| Maternal complications of pregnancy | 0.6% | 0.6% | 1.09 | 0.80 | 1.50 |
| Hepatobiliary neoplasms malignant and unspecified | 0.5% | 0.0% | NA |  |  |
| Hepatobiliary neoplasms | 0.5% | 0.0% | 1.99 | 1.14 | 3.46 |
| Dental and gingival conditions | 0.5% | 0.2% | 1.07 | 0.77 | 1.48 |
| Communication disorders and disturbances | 0.5% | 0.1% | 1.18 | 0.85 | 1.66 |
| Reproductive and genitourinary neoplasms gender unspecified NEC | 0.5% | 0.2% | 1.20 | 0.82 | 1.76 |
| Food intolerance syndromes | 0.5% | 0.2% | NA |  |  |
| Adrenal gland disorders | 0.5% | 0.1% | 1.11 | 0.77 | 1.61 |
| Male reproductive tract infections and inflammations | 0.5% | 0.4% | 1.73 | 1.27 | 2.38 |
| Maternal complications of labour and delivery | 0.5% | 0.6% | 0.82 | 0.58 | 1.18 |
| Immune system disorders congenital | 0.5% | 0.1% | 1.91 | 1.10 | 3.30 |
| Lymphatic vessel disorders | 0.5% | 0.0% | 2.20 | 1.37 | 3.54 |
| Breast neoplasms benign (incl nipple) | 0.4% | 0.8% | 0.53 | 0.38 | 0.73 |
| Hypothalamus and pituitary gland disorders | 0.4% | 0.2% | 0.87 | 0.58 | 1.31 |
| Neoplasm related morbidities | 0.4% | 0.0% | 2.03 | 1.28 | 3.22 |
| Congenital and peripartum neurological conditions | 0.4% | 0.1% | NA |  |  |
| Nervous system neoplasms benign | 0.4% | 0.1% | NA |  |  |
| Salivary gland conditions | 0.4% | 0.2% | 0.86 | 0.60 | 1.25 |
| Neurological disorders congenital | 0.4% | 0.1% | 1.45 | 0.96 | 2.19 |
| Skin vascular abnormalities | 0.4% | 0.1% | 0.75 | 0.50 | 1.14 |
| Renal and urinary tract neoplasms malignant and unspecified | 0.4% | 0.1% | NA |  |  |
| Aneurysms and artery dissections | 0.4% | 0.1% | 1.25 | 0.86 | 1.82 |
| Renal and urinary tract neoplasms benign | 0.4% | 0.1% | 0.67 | 0.45 | 1.01 |
| Ocular neuromuscular disorders | 0.4% | 0.2% | 1.41 | 0.92 | 2.16 |
| Eating disorders and disturbances | 0.4% | 0.1% | 0.89 | 0.59 | 1.33 |
| Ocular haemorrhages and vascular disorders NEC | 0.4% | 0.2% | 1.05 | 0.71 | 1.56 |
| Red blood cell disorders | 0.3% | 0.1% | 0.86 | 0.53 | 1.40 |
| Respiratory and mediastinal neoplasms benign (excl mesotheliomas) | 0.3% | 0.1% | 1.40 | 0.93 | 2.10 |
| Skin and subcutaneous tissue disorders congenital | 0.3% | 0.3% | 1.04 | 0.70 | 1.53 |
| Plasma cell neoplasms | 0.3% | 0.0% | 1.93 | 1.16 | 3.20 |
| Ectoparasitic disorders | 0.3% | 0.1% | 1.22 | 0.72 | 2.07 |
| Endocrine neoplasms benign | 0.3% | 0.1% | 0.47 | 0.27 | 0.82 |
| Abortions and stillbirth | 0.3% | 0.4% | 0.79 | 0.53 | 1.17 |
| Lymphomas non-Hodgkin's B-cell | 0.3% | 0.0% | NA |  |  |
| Lymphomas non-Hodgkin's unspecified histology | 0.3% | 0.1% | 0.81 | 0.42 | 1.56 |
| Blood and lymphatic system disorders congenital | 0.2% | 0.1% | 0.98 | 0.55 | 1.72 |
| Pericardial disorders | 0.2% | 0.0% | 1.20 | 0.62 | 2.32 |
| Injuries by physical agents | 0.2% | 0.1% | 1.14 | 0.71 | 1.83 |
| Suicidal and self-injurious behaviours NEC | 0.2% | 0.0% | 1.27 | 0.75 | 2.16 |
| Tongue conditions | 0.2% | 0.1% | 0.81 | 0.48 | 1.37 |
| Ocular injuries | 0.2% | 0.2% | 0.75 | 0.48 | 1.17 |
| Renal and urinary tract disorders congenital | 0.2% | 0.1% | 0.67 | 0.36 | 1.24 |
| Endocardial disorders | 0.2% | 0.1% | 0.81 | 0.43 | 1.52 |
| Lymphomas non-Hodgkin's T-cell | 0.2% | 0.0% | 2.43 | 1.17 | 5.04 |
| Reproductive tract and breast disorders congenital | 0.2% | 0.1% | 1.19 | 0.68 | 2.06 |
| Ocular neoplasms | 0.2% | 0.2% | 0.82 | 0.50 | 1.33 |
| Haemolyses and related conditions | 0.2% | 0.1% | 0.60 | 0.33 | 1.09 |
| Increased intracranial pressure and hydrocephalus | 0.2% | 0.0% | 2.09 | 1.14 | 3.84 |
| Postpartum and puerperal disorders | 0.2% | 0.1% | 1.47 | 0.85 | 2.54 |
| Congenital cardiac disorders | 0.1% | 0.1% | 1.03 | 0.54 | 1.96 |
| Ureteric disorders | 0.1% | 0.0% | 0.81 | 0.43 | 1.55 |
| Gastrointestinal tract disorders congenital | 0.1% | 0.1% | 0.53 | 0.27 | 1.04 |
| Personality disorders and disturbances in behaviour | 0.1% | 0.0% | 0.83 | 0.42 | 1.64 |
| Congenital reproductive tract and breast disorders | 0.1% | 0.1% | NA |  |  |
| Haemoglobinopathies | 0.1% | 0.1% | NA |  |  |
| Congenital eye disorders (excl glaucoma) | 0.1% | 0.1% | 0.53 | 0.27 | 1.03 |
| Skeletal neoplasms benign | 0.1% | 0.1% | 0.35 | 0.16 | 0.79 |
| Lymphomas Hodgkin's disease | 0.1% | 0.0% | 1.24 | 0.53 | 2.91 |
| Protozoal infectious disorders | 0.1% | 0.0% | 0.86 | 0.34 | 2.19 |
| Reproductive neoplasms male benign | 0.1% | 0.1% | 0.84 | 0.40 | 1.77 |
| Hepatobiliary disorders congenital | 0.1% | 0.0% | 0.80 | 0.30 | 2.09 |
| Chromosomal abnormalities and abnormal gene carriers | 0.1% | 0.1% | 1.01 | 0.46 | 2.22 |
| Impulse control disorders NEC | 0.1% | 0.0% | 1.27 | 0.51 | 3.16 |
| Respiratory disorders congenital | 0.1% | 0.0% | 0.62 | 0.16 | 2.37 |
| Endocrine disorders congenital | 0.0% | 0.0% | 0.47 | 0.17 | 1.26 |
| Vascular injuries | 0.0% | 0.0% | 1.37 | 0.46 | 4.05 |
| Chlamydial infectious disorders | 0.0% | 0.0% | 0.72 | 0.28 | 1.83 |
| Congenital respiratory tract disorders | 0.0% | 0.0% | 0.85 | 0.11 | 6.59 |
| Helminthic disorders | 0.0% | 0.0% | 1.38 | 0.36 | 5.27 |
| Mycobacterial infectious disorders | 0.0% | 0.0% | 0.42 | 0.11 | 1.58 |
| Dissociative disorders | 0.0% | 0.0% | 1.83 | 0.46 | 7.32 |
| Infections and infestations congenital | 0.0% | 0.0% | 23.55 | 1.60 | 346.29 |
| Hepatic and biliary neoplasms benign | 0.0% | 0.0% | 2.15 | 0.25 | 18.41 |
| Rickettsial infectious disorders | 0.0% | 0.0% | 1.01 | 0.14 | 7.36 |
| Developmental disorders NEC | 0.0% | 0.0% | 2.31 | 0.50 | 10.59 |
| Ear and labyrinthine disorders congenital | 0.0% | 0.0% | NA |  |  |
| Congenital ear disorders (excl deafness) | 0.0% | 0.0% | 0.54 | 0.07 | 4.52 |
| Mycoplasmal infectious disorders | 0.0% | 0.0% | 0.21 | 0.03 | 1.64 |
| Cardiac neoplasms | 0.0% | 0.0% | 0.00 | 0.00 | >999 |
| Cytoplasmic disorders congenital | 0.0% | 0.0% | 0.00 | 0.00 | >999 |

NA, not applicable, variable was not included in the logistic regression model due to a correlation coefficient >0.70 with at least one other variable.
